# Coagulopathy as initial manifestation of concomitant celiac disease and cystic fibrosis: a case report

**DOI:** 10.1186/1752-1947-5-116

**Published:** 2011-03-24

**Authors:** Aco Kostovski, Nikolina Zdraveska

**Affiliations:** 1 University Children's Hospital, Department for Gastroenterology and Hepatology, Skopje, Former Yugoslav Republic of Macedonia

## Abstract

**Introduction:**

Celiac disease and cystic fibrosis have many common manifestations, such as malabsorption, steatorrhea and growth failure, and were for many years recognized as one clinical entity. Since their recognition as two separate diseases, their co-existence in a patient has been described sporadically; around 20 cases have been described in the literature. Taking into consideration the incidences of the two diseases, the chance of them occurring together is one in 2,000,000 in the general population.

**Case presentation:**

We describe the case of a five-year-old boy of Turkish ethnicity with both celiac disease and cystic fibrosis, who presented initially with a skin hemorrhage. The diagnosis of celiac disease was made with a positive serum anti-tissue transglutaminase antibody test and the presence of HLA-DQ2 heterodimer, and confirmed on histology with small intestinal villous atrophy. A positive sweat test confirmed the diagnosis of associated cystic fibrosis.

To the best of our knowledge there has been no previous report of this rare presentation of associated celiac disease and cystic fibrosis.

**Conclusion:**

The clinical significance of this case is the consideration of malabsorption with both celiac disease and cystic fibrosis in patients who present with unexplained coagulopathy.

## Introduction

Celiac disease (CD) is a multi-factorial, autoimmune disorder that occurs in genetically susceptible individuals, triggered by a well-identified environmental factor--gluten. Originally considered to be a rare malabsorption syndrome of childhood, CD is now recognized as a common condition that may be diagnosed at any age and that affects many organ systems. The interplay between genes and the environment leads to the onset of intestinal and/or extraintestinal symptoms.

Cystic fibrosis (CF) is the most common genetically inherited disease in Caucasian populations. Understanding of the disease has progressed rapidly the past 20 years. CF used to be predominantly a lung disease of young children but more recently it has become a complex multi-system disease extending into adulthood. The co-existence of CD and CF was first described, in a child, by Hide and Burman in 1969 [1], and around 20 cases have been reported in the literature.

We present the case of a boy with concomitant CD and CF, initially manifesting with a skin hemorrhage. To the best of our knowledge there has been no previous report of this rare hemorrhagic presentation of concomitant CD and CF.

### Case report

A five-year-old boy of Turkish ethnicity was referred to our hospital because of the spontaneous appearance of hematomas on both his upper and lower limbs and on his back over the preceding few weeks. His parents were healthy, unrelated, and he had no siblings. No family history for genetic diseases was reported. He was born out of normal pregnancy and delivery, with a birth weight of 4.1 kg; had normal peri-natal course and regular weight gain. He was barely breastfed and cow's milk was introduced from his first month. There were no previous hospital admissions. He did not have any major illnesses except for a few upper respiratory infections which were successfully treated with medication. In addition to these, he had experienced occasional, mild, non-troublesome cough that had never required treatment. His abdomen was always distended and his mother reported three to four loose stools per day in the last five months.

A general physical examination on admission revealed skin paleness, but no jaundice, and remarkable multiple bruises on both his legs and right arm and a single large hematoma in the lumbar region of his back. An examination of his abdomen showed marked abdominal distension, with no hepatosplenomegaly and no palpable mass or ascites. His height was on the third percentile, his weight was within the 25^th ^percentile for his age. His body mass index was 16.5 (within the 50^th ^percentile for his age).

A laboratory analysis was suggestive of iron deficiency anemia, with hemoglobin level 97 g/L, serum iron 2.8 μmol/L, hypoproteinemia (50 g/L protein), hypoalbuminemia (23 g/L albumin), elevated serum aspartate transaminase (AST) (129 U/L), and elevated alanine transaminase (ALT) (159 U/L). A coagulation screening profile showed normal platelets number and bleeding time, but a prolonged prothrombin time (PT) of 63s (normal range 12 to 15s), and prolonged activated partial thromboplastin time (aPTT) of 105s (normal 34s). Possible primary liver disease was excluded, with negative antibodies for viral hepatitis A, B and C, as well as negative antibodies for autoimmune hepatitis type I and II. Ceruloplasmin and α1-antitrypsin were within normal limits for his age. A liver biopsy was not performed because of the severe coagulation impairment. A rapid screening test for tissue transglutaminase IgA antibodies was positive, with quantitative measurements of 903.3IU/mL (normal range 20IU/mL). Antigliadin IgA was 1822IU/mL (normal range < 25IU/mL) and antigliadin IgG was 922.6IU/mL (normal range <25IU/mL). Histologic assessment of the intestine revealed villous atrophy, cryptal elongation and hyperplasia plus increased intra-epithelial lymphocytes and mononuclear cells infiltration into the lamina propria. HLA typing was positive for HLA-DQ2 (DQB1*02 homozygous). A sweat test was preformed on two separate occasions and came back positive, with chloride concentration measuring 118 mmol/L and 67 mmol/L. A chest radiography showed hyperinflation with increased retrosternal air space and reduced transparency in his lower lung lobes, especially expressed on the right side. The molecular work up was negative for the most frequent mutations of cystic fibrosis transmembrane conductance regulator (CFTR) (only 11 are available in our center). Our patient was diagnosed to have both CD and CF. Treatment with ursodeoxycholic acid and pancreatic enzymes, antibiotics and oral iron supplementation was initiated and a gluten-free diet was recommended.

During the hospital stay plasma transfusion and Vitamin K substitution was administered to normalize the coagulation tests, as well as albumin supplementation. The PT and aPTT returned to normal values and remained normal throughout the rest of his hospitalization. Our patient was followed-up two months after the treatment, during which he was on a gluten-free diet and oral iron supplementation. The child looked healthy and had gained weight; his stools became less frequent with normal consistency. No recurrent bleeding or hematomas were detected. His liver enzymes, serum protein and albumin values were normal. Coagulation tests were normal without additional vitamin K supplementation. A sweat test was performed again, and was positive with chloride concentration of 72 mmol/L.

### Discussion

CD is an autoimmune inflammatory enteropathy that is triggered by the ingestion of gluten-containing grains in genetically susceptible individuals. CD is one of the most common lifelong disorders on a worldwide basis, affecting nearly 1% of the general population in the USA and other developed countries [2]. Studies in the last years have shown that the prevalence of CD has substantial increased [3]. The availability of sensitive and specific serological tests has made it possible to assess the true prevalence of CD by detecting the minimally symptomatic or asymptomatic cases with typical mucosal changes. Results from genetic linkage studies have shown that CD is strongly associated with HLA-DQ genes located on chromosome 6p21. Approximately 90-95% of patients with CD present DQ2 heterodimer and most of the remaining cases carry HLA-DQ8 molecules. The absence of these alleles is important for their high negative predictive value [4,5].

The clinical spectrum of CD is wide, including cases with either typical intestinal features or atypical extraintestinal features. Recently, attention has been focused on the atypical presentations of CD, such as isolated anemia, osteoporosis, short stature, peripheral neuropathy, rickets, constipation, or delayed puberty. Whilst these presentations of CD have become more recognized, hemorrhagic presentations of CD owing to coagulopathy are quite rare. It can result from vitamin K deficiency due to malabsorption or as a consequence of liver injury [6].

Persistent elevation of serum aminotransferase activity is the most common liver abnormality found in patients with CD and may occur in up to 60% of cases. This is a reversible gluten-related type of liver damage known as celiac hepatitis [7]. Severe hepatic damage rarely occurs in patients with CD. Casswall *et al*. reported six children treated in their center with acute liver failure and coagulopathy associated with CD over a 12 year period of time. Two of them had to receive liver transplants. Thus, they suggested routinely checking for liver function in all children with new onset CD, and also investigating for untreated CD in children with severe liver damage [8].

CF is the most common autosomal recessive disease, with a global incidence of 1 in 2500 newborns. The gene responsible for this disease--CFTR--was identified in 1989. The most common CFTR defect is the ΔF508 mutation, occurring in about 70% of patients with CF [9]. Currently more than 1600 mutations in the CFTR gene have been described. Different CFTR mutations result in different disease phenotypes. Some may have little or no effect on CFTR function and may result in milder forms of disease. According to the generally accepted criteria, diagnosis of CF requires one or more characteristic clinical features in combination with laboratory evidence of CFTR dysfunction (two elevated sweat chloride concentrations obtained on separate days) or identification of two pathologic CFTR mutations [10].

Unlike pulmonary and pancreatic manifestations that are present in 80-90% of CF patients, liver involvement is much less frequent, affecting only one third of CF patients [11]. Corrigan *et al. *in 1981 documented a coagulopathy in 17 (71%) of 24 patients with CF, mean age 17 years. In three cases this was suggestive of liver disease, and in 14, of vitamin K deficiency [12]. In 1994 Durie assessed the need for routine vitamin K supplementation in patients with CF [13]. Factors in CF that predispose patients to vitamin K deficiency may include malabsorption, cholestatic or non-cholestatic liver disease, and chronic antibiotic intake [14].

CD and CF share a number of clinical manifestations and were for many years recognized as one clinical entity [9]. Co-existence of the two diseases in the same patient has been reported sporadically since the 1960s. In 1999, Venuta *et al. *described a patient suffering from CD and CF and reviewed the available literature, summarizing 16 documented cases of CD co-existing with CF [15]. Valletta and Mastella in 1989 described five CD cases among 1100 CF patients and the incidence of CD co-morbidity was calculated to be at least one in 220 (0.45%) [16]. Fluge *et al. *in 2009 performed a systematic screening for CD in a large Scandinavian cohort of 790 CF patients, and detected 10 patents with CD (1.2%) [17]. A recent study from Poland reported 2.13% incidence of CD among 230 CF patients [18].

Taking into consideration the incidences of these two diseases, the chance of their occurring together in the general population is one in 2,000,000 [19].

In the literature there are some hypotheses to explain the co-existence of CD and CF. Due to pancreatic insufficiency in patients with CF, the mucosa of the bowel may have more contact with the complete gluten protein. In addition, malnutrition might contribute to some additional mucosal damage [18]. Some studies also suggest increased incidence of food allergy in CF patients [19].

In our case, the first challenge was to identify the etiology of the bleeding diathesis. The abnormality of both the PT and PTT, as seen in our patient, led to the consideration of a multiple coagulation factors abnormality. In such abnormalities the hepatic synthesis of factors II, VII, IX, and X from vitamin K is impaired. The most common manifestations, bleeding from hypoprothrombinemia, are ecchymosis and hematomas, as seen in our patient. Vitamin K malabsorption leading to hemorrhage is an unusual presentation of both CD and CF. The chronic diarrhea, that had not been diagnosed until this episode, assisted in narrowing the diagnosis. The positive serologic test in correlation with HLA-DQ2 presence was strongly suggestive for CD. Intestinal histology and marked clinical improvement after gluten withdrawal confirmed the diagnosis. Our patient also fulfilled the diagnostic criteria for CF because of the manifested pancreatic insufficiency and a chest radiography, with the initial diagnosis confirmed with the positive sweat test.

## Conclusion

The clinical significance of this case is in the consideration CD and CF in patients who present with unexplained coagulopathy. The presence of abnormal tests of coagulation should prompt the clinician to consider any signs of malabsorption. The co-existence of CD and CF in this case was also a motive to systematically screen for CD among other CF-diagnosed patients in our center.

## Consent

Written informed consent was obtained from the patient's father for publication of this case report and any accompanying images. A copy of the written consent is available for review by the Editor-in-Chief of this journal.

## Competing interests

The authors declare that they have no competing interests.

## Authors' contributions

AK analyzed and interpreted the patient data and performed the GI endoscopy. NZ analyzed and compiled all the data and laboratory analysis and was the major contributor in writing the manuscript. All authors read and approved the final manuscript.
